# Medical Association of Central New York

**Published:** 1895-12

**Authors:** 


### Society Proceedings.

### MEDICAL ASSOCIATION OF CENTRAL NEW YORK.

THE twenty-eighth annual meeting of the Medical Association of Central New York was held in the assembly-room of the Yates hotel, in the city of Syracuse, N. Y., Tuesday, October 15, 1895.

The association was called to order at eleven o'clock by the president, Dr. FLOYD S. CREGO, of Buffalo. The reading of the minutes of last meeting was dispensed with, and, upon motion, they were approved as printed in the proceedings.

The PRESIDENT then announced the following committees :

*Credentials*—Drs. A. A. Young, of Wayne ; S. F. Snow, of Onondaga ; H. T. Carter, of Erie.

*Arrangement and Reception*—Drs. E. L. Mooney, H. L. Elsner and A. C. Mercer, of Onondaga.

*Business*—Drs. F. H. Stephenson, of Onondaga ; A. L. Beahan, of Ontario ; John Gerin, of Cayuga.

The publication committee reported that the arrangement with the BUFFALO MEDICAL JOURNAL for publishing the transactions had proven satisfactory, and that a copy of the volume containing the minutes, papers and discussions of the last meeting had been sent to each member who had paid the annual dues. It had been deemed best not to publish the revised constitution and list of members during the past year, but the committee advised that this be done during the current year.

The report was accepted and the publication committee was directed to publish the revised constitution and list of members, and mail a copy to each member.

The Secretary read a list of delegates who had registered at the meeting in Buffalo last year who by attendance at the meeting this year were entitled to membership.

The vice-president, Dr. W. S. Cheesman, of Cayuga, then took the chair while the president, Dr. Floyd S. Crego, delivered the annual address.

Upon motion, a vote of thanks was extended by the association to the president for his excellent address.

Dr. Stephenson, as chairman of the business committee, then announced that twenty minutes would be allowed for each paper, unless the discussions were prolonged, in which case the president would reduce later the time allotted.

Papers were then read as follows : 1. Fracture of the greater tuberosity of the humerus—frequency, cause, complication, diagnosis, treatment and result, with report of a case, by Dr. A. L. Hall, of Fair Haven, N. Y. ; discussed by Dr. Jacobson, of Syracuse. 2. Aural, nasal and laryngeal tuberculosis—with special reference to the Adirondacks as a winter health resort, by Dr. Sargent F. Snow, of Syracuse, N. Y. ; discussed by Drs. Halstead, Creveling, Elsner, Herriman and the president. 3. Some observations in general paresis, by Dr. F. H. Stephenson, of Syracuse, N. Y. 4. Report of a case of appendicitis without preceding pain or fever, by Dr. S. L. Elsner, of Rochester, N. Y., which was later discussed by Drs. Cheesman, Jacobson, Baker, Miller and S. L. Elsner.

The Secretary then announced the following list of delegates who had complied with the requirements of the by-laws, and moved their election to permanent membership : Drs. Henry T. Carter, of Erie ; B. C. Loveland, Alfred M. Mead and C. O. Jackson, of Ontario ; Eugene H. Carpenter, of Madison ; T. Oliver Tait, Henry H. Covell, John Zimmer, Charles A. Vanderbeek and W. D. Wolff, of Monroe. Carried.

Dr. Bacon, of Oswego county, offered the following resolution :

*Resolved*, That it is the sense of the association that the insuring of the lives of children under ten years of age, as at present conducted, is against public policy.

That we recommend to our law makers to pass a law that shall prohibit a practice in this country that has proved so dangerous in other parts of the world.

Upon motion the resolution was referred to a committee to report after luncheon.

The PRESIDENT appointed Drs. C. C. Bacon, of Oswego, Charles A. Vanderbeek, of Monroe, and J. P. Creveling, of Cayuga, as such committee.

The meeting was then adjourned to 2 o'clock P. M., and luncheon was served in the dining-room of the Yates hotel by the invitation of the Onondaga County Medical Society.

The association reassembled at 2.30 o'clock. The committee appointed to consider the resolution offered by Dr. Bacon desired an extension of time, and upon motion the committee was instructed to report at the next annual meeting.

The report of the TREASURER was read and showed a balance on hand of $182.05.

The SECRETARY announced that he had some copies of the transactions left over from last year, and it had been decided to let the members and delegates have them at cost price, fifty cents a copy. They could be obtained upon application to the secretary.

The election of officers for the ensuing year resulted as follows: President, Dr. William S. Cheesman, of Auburn; first vice-president, Dr. Edward B. Angell, of Rochester; second vice-president, Dr. E. L. Mooney, of Syracuse; secretary, Dr. Charles A. Vanderbeek, of Rochester; treasurer, Dr. S. L. Elsner, of Rochester.

The SECRETARY presented his bill for printing and other expense items, amounting to $43.15, which was ordered paid.

Secretary announced the following visitors: B. W. Loomis, of Syracuse; E. B. Mervin, of Camillus; H. D. Mervin, of Cicero; S. H. Levy and C. F. Wright, of Syracuse; F. F. Cooley, of Oswego; N. Wilbur, of Fayetteville; E. W. Belknap, C. N. Daman and C. E. McClary, of Syracuse; C. E. Heaton, of Baldwinsville; Charles E. Weidman, of Marcellus; A. W. Scott, of Bridgeport; Irving M. Snow and F. C. Busch, of Buffalo.

The PRESIDENT then appointed the following committee on publication for the ensuing year: Drs. E. B. Angell and Charles A. Vanderbeek, of Monroe and Dr. William C. Krauss, of Erie.

The following papers were then read: 5. Demonstration of landmarks of stomach, liver and the like, by auscultatory percussion, by Dr. A. L. Benedict, of Buffalo. 6. Localised peritonitis, by Dr. C. O. Baker, of Auburn, N. Y. 7. Treatment of ileocolitis in the Buffalo Fresh Air Hospital, by Dr. Irving M. Snow, of Buffalo. 8. A pseudencephalic monster, with exhibition of specimen, by Dr. Wm. L. Conklin, of Rochester, N. Y. 9. The relation of diseased conditions of the nose and throat to malnutrition, by Dr. John O. Roe, of Rochester, N. Y.; discussed by Drs. Snow, Didama, Loveland and Roe.

The following papers were read by title: 10. Concerning the record of cases, by Dr. Lucien Howe, of Buffalo, N. Y. 11. Amebic catarrh of the intestinal tract, by Dr. A. A. Young, of Newark, N. Y. 12. Exophthalmic goitre, by Dr. W. C. Krauss, of Buffalo, N. Y.

The following papers were then read: 13. Vaginal celiotomy, by Dr. Wm. B. Jones, of Rochester, N. Y.; discussed by Dr. Miller, of Syracuse. 14. The relation between the specific gravity of the blood and its hemoglobin percentage, by Mr. A. T. Kerr, Jr., of Buffalo, N. Y.

Dr. A. A. Young moved that in view of the excellent entertainment by the Onondaga County Medical Society we tender them a vote of thanks. Carried.

President Crego: It is the unanimous sentiment of the society that we have been royally entertained.

The committee of arrangements was then authorized to draw upon the treasurer for extraordinary expenses to the amount of $25, if necessary.

The association then adjourned to meet at Rochester, the third Tuesday in October, 1896.

